# Comparison between Machine Learning and Multiple Linear Regression to Identify Abnormal Thallium Myocardial Perfusion Scan in Chinese Type 2 Diabetes

**DOI:** 10.3390/diagnostics12071619

**Published:** 2022-07-03

**Authors:** Jiunn-Diann Lin, Dee Pei, Fang-Yu Chen, Chung-Ze Wu, Chieh-Hua Lu, Li-Ying Huang, Chun-Heng Kuo, Shi-Wen Kuo, Yen-Lin Chen

**Affiliations:** 1 Division of Endocrinology, Department of Internal Medicine, Shuang Ho Hospital, Taipei Medical University, New Taipei City 23651, Taiwan; d118102003@tmu.edu.tw (J.-D.L.); chungze@tmu.edu.tw (C.-Z.W.); 2 Division of Endocrinology and Metabolism, Department of Internal Medicine, School of Medicine, College of Medicine, Taipei Medical University, Taipei City 11031, Taiwan; 3 School of Medicine, College of Medicine, Fu Jen Catholic University, New Taipei 24205, Taiwan; a00119@mail.fjuh.fju.edu.tw (D.P.); a02950@mail.fjuh.fju.edu.tw (F.-Y.C.); a00112@mail.fjuh.fju.edu.tw (L.-Y.H.); a01106@mail.fjuh.fju.edu.tw (C.-H.K.); 4 Division of Endocrinology and Metabolism, Department of Internal Medicine, Fu Jen Catholic University Hospital, New Taipei 24352, Taiwan; 5 Division of Endocrinology and Metabolism, Department of Internal Medicine, Tri-Service General Hospital, School of Medicine, National Defense Medical Center, No. 325. Sec. 2, Chenggong Rd., Neihu District, Taipei City 11490, Taiwan; 897010010@mail.ndmctsgh.edu.tw; 6 Division of Endocrinology and Metabolism, Department of Internal Medicine, Taipei Tzu Chi Hospital, Buddhist Tzu Chi Medical Foundation, No. 289, Jianguo Rd., Xindian Dist., New Taipei City 23142, Taiwan; 7 Department of Pathology, Tri-Service General Hospital, National Defense Medical Center, No. 325. Sec. 2, Chenggong Rd., Neihu District, Taipei City 11490, Taiwan

**Keywords:** type 2 diabetes mellitus, coronary artery disease, thallium-201 myocardial perfusion scan, machine learning

## Abstract

Type 2 diabetes mellitus (T2DM) patients have a high risk of coronary artery disease (CAD). Thallium-201 myocardial perfusion scan (Th-201 scan) is a non-invasive and extensively used tool in recognizing CAD in clinical settings. In this study, we attempted to compare the predictive accuracy of evaluating abnormal Th-201 scans using traditional multiple linear regression (MLR) with four machine learning (ML) methods. From the study, we can determine whether ML surpasses traditional MLR and rank the clinical variables and compare them with previous reports.In total, 796 T2DM, including 368 men and 528 women, were enrolled. In addition to traditional MLR, classification and regression tree (CART), random forest (RF), stochastic gradient boosting (SGB) and eXtreme gradient boosting (XGBoost) were also used to analyze abnormal Th-201 scans. Stress sum score was used as the endpoint (dependent variable). Our findings show that all four root mean square errors of ML are smaller than with MLR, which implies that ML is more precise than MLR in determining abnormal Th-201 scans by using clinical parameters. The first seven factors, from the most important to the least are:body mass index, hemoglobin, age, glycated hemoglobin, Creatinine, systolic and diastolic blood pressure. In conclusion, ML is not inferior to traditional MLR in predicting abnormal Th-201 scans, and the most important factors are body mass index, hemoglobin, age, glycated hemoglobin, creatinine, systolic and diastolic blood pressure. ML methods are superior in these kinds of studies.

## 1. Introduction

Type 2 diabetes (T2DM) is the fifth most common cause of death in Taiwan and its prevalence has been increasing drastically over the last three decades [1]. Now in Taiwan, there are 2.457 million T2DM which is 9.7% of the total population [2]. Similarly, it is expected that there will be 700 million patients throughout the world by 2045 [3]. In the past, having T2DM would reduce life expectancy by around six years compared to healthy comparators [4]. Evidence has shown that approximately 50% of T2DM patients die of cardiovascular disease [5,6]. At the same time, evidence has shown that T2DM increases the risk of coronary artery disease (CAD) and that T2DM is associated with a 70% excess risk of acute myocardial infarction (MI) [7,8,9]. More importantly, 28.5% of the MI is silent in T2DM [10]. Therefore, early detection of CAD in diabetic patients is important for health providers. Guidelines have been developed to define a high-risk profile of diabetic patients who might benefit from routine cardiac screening [11,12].

Several examinations are used to evaluate the status of CAD. For instance, coronary angiography is considered the “gold standard”, but it is invasive and has attendant risks. The second one, computed tomography coronary angiography (CTCA), although non-invasive, is relatively expensive. The third commonly used test is exercise electrocardiogram (ECG). However, this can only be used for patients who can exercise to a sufficient workload [13,14]. Lastly, pharmacologic stress testing with myocardial perfusion scintigraphy (MPS) using Thallium as a tracer is also non-invasive in nature. In diabetic patients, it has been shown to be of value for diagnosing significant CAD, stratifying risk and future management [15]. This examination has been well-accepted as a tool to identify patients with CAD and predicting CAD prognosis in clinical settings. Since MPS could be taken as a surrogate for CAD, there are studies trying to identify risk factors for abnormal MPS [16]. Several risk factors have been identified as being associated with abnormal MPS, including current smoking, duration of diabetes and the cholesterol/high-density lipoprotein (HDL) ratio, etc. It should be noted that all these studies used traditional multiple linear regression (MLR) to analyze the data.

Since the rapid progress of computational facilities, artificial intelligence using machine learning (ML) has developed rapidly and has been used in some of the research areas in the medical field, including cancer, cardiovascular disease, neurological disease, emergency medicine and even in the pharmacological field, etc. [17,18,19,20,21]. The definition of ML is the study of computer algorithms that can improve automatically through experience and by the use of data [22]. It enables machines to learn from past data or experiences without being explicitly programmed. After certain computer algorithms are created using the ML method, the process has many parameters to predict future results. It now becomes a new modality for data analysis competitive with traditional MLR [23,24,25]. Since ML could capture nonlinear relationships in the data and complex interactions among multiple predictors, it has the potential to outperform conventional logistic regression in disease prediction [26].

The present study was performed with a T2DM cohort without diagnosed CAD and there were two aims: 1. To compare whether ML is more accurate than MLR. 2. To rank the risk factors and compare their orders to previous reports.

## 2. Materials and Methods

### 2.1. Subjects

**Figure 1:**
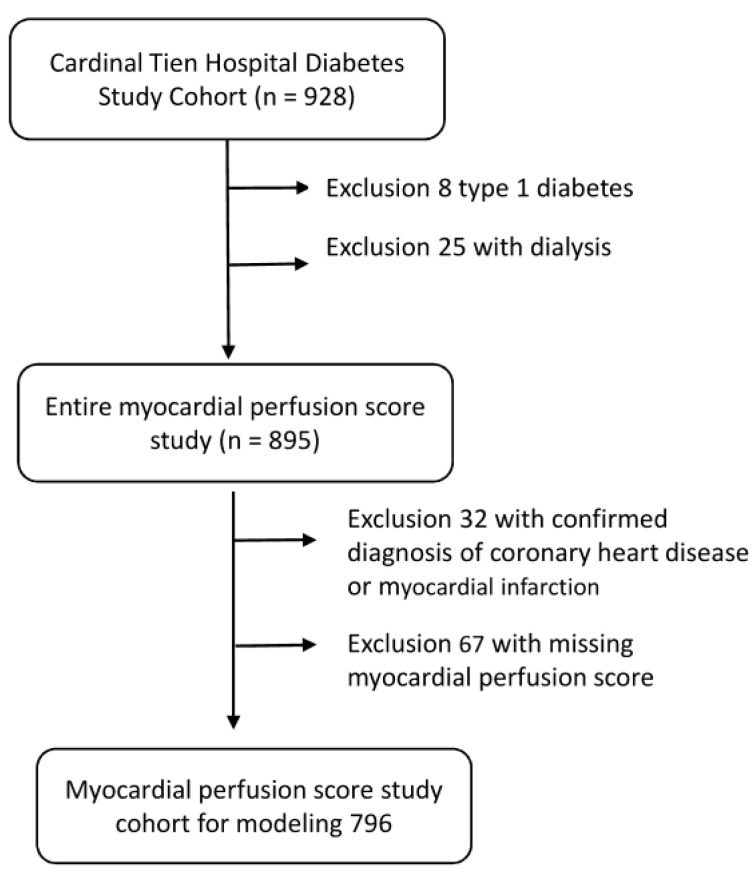
Flowchart of sample selection from the Cardinal Tien Hospital Diabetes Study Cohort.

T2DM patients, aged between 30 and 95 years old, who had undertaken Thallium-201 myocardial perfusion scans (Th-201 scan) in Cardinal Tien hospital from 1999 to 2008 were recruited for the study. All study subjects were anonymous and the data of the participants were used only for the analysis. This is a retrospective study, and all the data were retrieved from medical records from the hospital. The study proposal was reviewed and approved by the institutional review board of Cardinal Tien hospital before the study began. On the day of the thallium scan, a thallium scan consent form provided by the Nuclear Medicine Department woud be obtained from the individual who received the examination. The diagnostic criteria for T2DM were based on the 2012 American Diabetes Association criteria [27]. In total, 928 T2DM patients were recruited. After some subjects were excluded due to various causes, 796 patients remained for analysis, including 368 men and 428 women. Figure 1 illustrates the flowchart of the subject selection in the present study.

Body mass index (BMI) was calculated as body weight (kg)/height (m)^2^. Systolic and diastolic blood pressure (SBP and DBP) were measured on the right arm of seated subjects using a standard mercury sphygmomanometer. Blood samples were drawn from the antecubital vein for biochemical analysis.

### 2.2. Th-201 Scan

On the day of testing, patients fasted for 4 h and withheld dipyridamole, β-blockers, calcium channel blockers, long-acting nitrates, xanthine-containing medications and caffeine-containing beverages. Each patient then received intravenous infusion of dipyridamole over 4 min at a concentration of 0.56 mg/kg in 20 mL of normal saline (an infusion rate of 0.14 mg/kg/min). Th-201 was administered intravenously 3 to 4 min after the dipyridamole infusion was completed. The scans started at 5 to 8 min after radiopharmaceutical administration (stress scan) and 3 h later (rest scan).

The myocardial region was classified into 17 parts and each part was evaluated by nuclear medicine experts based on a 5-point scoring system described previously [28]: 0, normal; 1, slight decrease of tracer uptake; 2, moderate decrease of tracer uptake; 3, severe decrease of tracer uptake; 4, absence of tracer uptake. The stress score and rest score of single vessels were initially counted as individual vessel scores. The sums of individual vessel stress scores (after injection of dipyridamole) were recognized as representative of the Th-201 results (dependent variable) since some of the studies have shown that SSS provides important information for detecting CAD and its outcomes [28,29,30].

### 2.3. Laboratory Evaluation

After the 10 h overnight fast, blood specimens were collected from each subject for further analysis. Plasma was separated from the whole blood within one hour and stored at −70 °C. A glucose oxidase method (YSI 203 glucose analyzer; Scientific Division, Yellow Springs Instruments, Yellow Springs, OH, USA) was used to determine fasting plasma glucose (FPG) levels. The dry, multilayer analytical slide method of the Fuji Dri-Chem 3000 analyzer (Fuji Photo Film, Minato-Ku, Tokyo, Japan) was used to determine total cholesterol and triglyceride (TG). An enzymatic cholesterol assay following dextran sulfate precipitation was used to determine serum HDL-C and low-density lipoprotein cholesterol (LDL-C) levels. The HbA1c level was measured using the Bio-Rad Variant II automatic analyzer (Bio-Rad Diagnostic Group, Los Angeles, CA, USA). Plasma insulin was assayed using a commercial solid-phase radioimmunoassay technique (Coat-A-Count insulin kit, Diagnostic Products Corporation, Los Angeles, CA, USA) with intra- and inter-assay coefficients of variance of 3.3% and 2.5%, respectively.

### 2.4. Statistical Analysis:

The data were tested for normal distribution using the Kolmogorov–Smirnov test and for homogeneity of variances using the Levene’s test. Continuous variables were expressed as mean ± standard deviation.

Table 1 lists the definition of the fifteen baseline clinical variables (independent variables, sex, age, BMI, duration of diabetes, smoking, FPG, glycated hemoglobin (HbA1c), TG, HDL-C, LDL-C, alanine aminotransferase (ALT), creatinine (Cr), microalbuminuria, SBP and DBP, used in this study. As aforementioned, the SSS derived from the Th-201 scan is the dependent variable; the other 15 variables are used as predictor variables.

### 2.5. ML Methods and Proposed Scheme

This research proposed a scheme based on four ML methods, namely classification and regression tree (CART), random forest (RF), stochastic gradient boosting (SGB) and eXtreme gradient boosting (XGBoost) to construct predictive models for determining abnormal MPS and to identify the importance of these risk factors. These ML methods have been widely applied to various healthcare and/or medical informatics applications and do not have prior assumptions about data distribution [31,32,33,34,35,36,37,38,39]. MLR is used as a benchmark for comparison.

The first method, CART, is a tree structure method [40]. It is composed of root nodes, branches and leaf nodes that, based on tree structures, grow recursively from the root nodes and split at each node based on the Gini index to produce branches and leaf nodes using the rule. The nodes of overgrown trees are then pruned for optimal tree size using cost–complexity criterion, and different decision rules are generated to compose a complete tree [41,42].

RF, the second method in this study, is an ensemble learning decision trees algorithm which combines bootstrap resampling and bagging [43]. RF’s principle method is to randomly generate many different and unpruned CART decision trees for which decreased Gini impurity is regarded as the splitting criterion, and then to combine all the trees generated into a forest. Then all the trees in the forest are averaged or voted to generate output probabilities and a final robust model [44].

The third method, SGB, is a tree-based gradient boosting learning algorithm combining both bagging and boosting techniques to minimize the loss function and solve the over-fitting problem of traditional decision trees [45,46]. The SGB sequentially and stochastically generates many weak learner trees through multiple iterations and each tree concentrates on correcting or explaining the errors from the tree of the previous iteration generated. The residual of the previous iteration tree is used as the input for the newly generated tree. This iterative process continues until a stopping criterion is reached at the maximum number of iterations or the convergence condition. The cumulative results of many trees are used to determine a final robust model.

XGBoost, the fourth method in this study, is gradient boosting technology based on SGB optimized extension [47]. Its principle is training many weak models sequentially to ensemble them using the gradient boosting method of outputs to achieve better prediction performance. In XGBoost, the Taylor binomial expansion is used to approximate the objective function and arbitrary differentiable loss functions to accelerate the model construction converging process [48]. Then, XGBoost applies a regularized boosting technique to reduce the complexity of the model and correct the overfitting, thus increasing the model accuracy [47].

**Figure 2:**
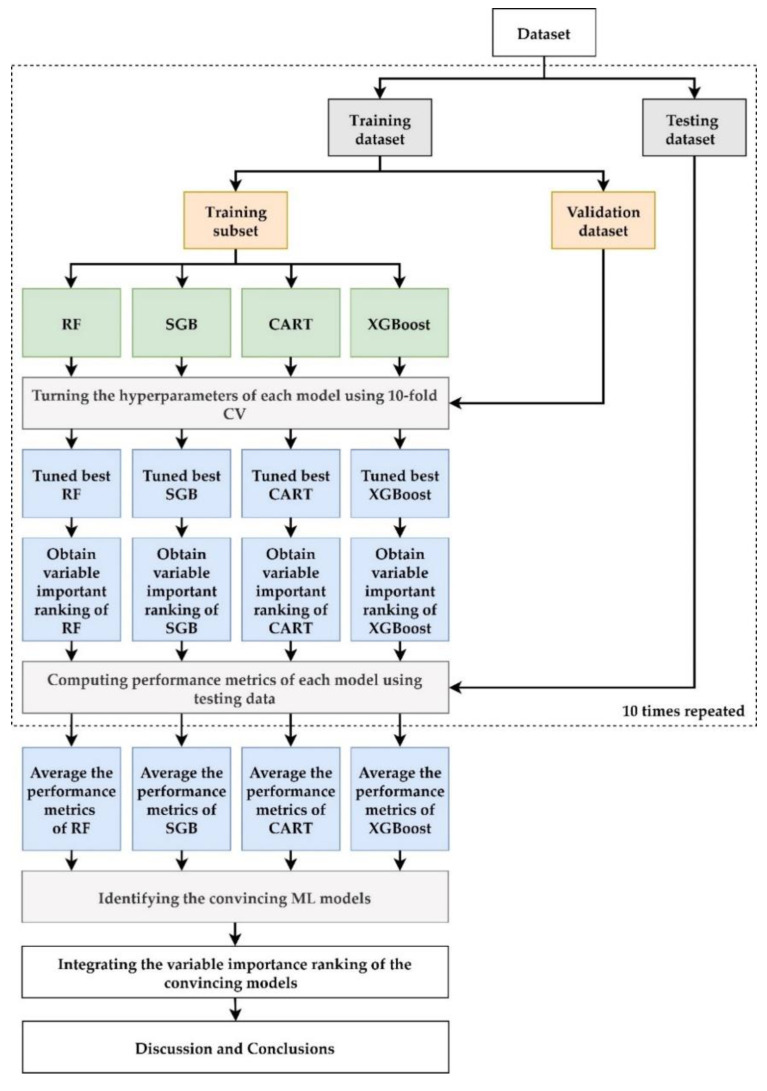
Proposed scheme in the study.

The flowchart of the proposed scheme combining the four ML methods is demonstrated in Figure 2. As Figure 2 shows, in the proposed scheme, we first collected patients to prepare the dataset for model construction, and then the dataset was randomly split into 80% training dataset for model building and 20% testing dataset for out-of-sample testing. In the training process, each ML method had its own hyperparameters to be tuned for constructing a relatively well-performing model. We used a 10-fold cross-validation (CV) technique for hyperparameter tuning. To do this, the training dataset was further randomly divided into the training dataset to build the model with a different set of hyperparameters, and the validation dataset for model validation. All possible combinations of hyperparameters were investigated using grid search. The model with the lowest root mean square error on the validation dataset was viewed as the best model of each ML method. The best models of RF, SGB, CART and XGBoost were generated and the corresponding variable importance ranking was obtained.

During the testing process, the testing data set is used to evaluate the predictive performance of the best ML models. The metrics used for model performance comparison are symmetric mean absolute percentage error (SMAPE), root mean square error (RMSE), root-relative square error (RRSE), and relative absolute error (RAE), which are shown in Table 2.

The metrics of RF, SGB, CART and XGBoost models were used to compare the model performance of the benchmark MLR model which used the same training and testing dataset as the ML methods. An ML model with an average metric lower than that of MLR was considered a convincing model. In order to evaluate whether the ML methods outperform MLR, after ML methods were repeated 10 times, means and standard deviations were obtained.

As all of the used ML methods can produce an importance ranking for each predictor variable, we defined that the variable ranked 1 would be the most critical risk factor and the variable ranked as 15 would be the least significant risk factor. The different ML methods may produce different variable importance rankings since they have different modeling characteristics; we integrated the variable importance ranking of the convincing ML models to enhance stability and integrity by re-ranking the importance of risk factors. In the final stage of the proposed scheme, we summarized and discussed our significant findings of convincing ML models and identified important variables.

In this study, all methods were performed with R software version 4.0.5 and R Studio version 1.1.453 with the required packages installed (http://www.R-project.org; https://www.rstudio.com/products/rstudio/ assessed 1 May 2022). The implementations of RF, SGB, CART and XGBoost are, respectively, “random Forest” R package version 4.6-14 [49], “gbm” R package version 2.1.8 [50], “r part” R package version 4.1-15 [51], “XGBoost” R package version 1.5.0.2. [52]. To estimate the best hyperparameters set for developed effective CART, RF, SGB and XGBoost methods, the “caret” R package version 6.0-90 was used [53]. The MLR was implemented by the “stats” R package version 4.0.5, the default setting was used to construct the models.

## 3. Results

The demographic data of the enrolled T2DM patients are shown in Table 3. Table 4 displays the comparison between conventional MLR and the four ML methods in identifying abnormal Th-201, and we found that all four ML methods exhibited low prediction errors compared to o the MLR method. These findings suggest that all ML methods are reliable and not inferior to traditional MLR. In order to further determine whether the four ML methods significantly outperformed the MLR method, the Wilcoxon signed-rank test was used. We used the test to evaluate the prediction performance of the four ML methods and the MLR method (data not shown). It can be observed that the prediction error values of all ML methods were not significantly different from the MLR method.

The ranking of each factor created by ML is demonstrated in Table 5. Diverse ML methods generated different relative importance rankings for each risk factor. Note that the darkness of the blue color indicates the importance of the risk factors. The darker the blue color is, the more important the risk factor is. For instance, in the SGB method, the top three important factors are BMI, HbA1c and Cr. For the CART method, the most dominant factor is BMI, followed by Cr and age. In addition, to identify the overall predictive power of each parameter from all four ML methods, the mean ranking of each risk factor was obtained by averaging the ranking values of each variable in each method.

**Figure 3:**
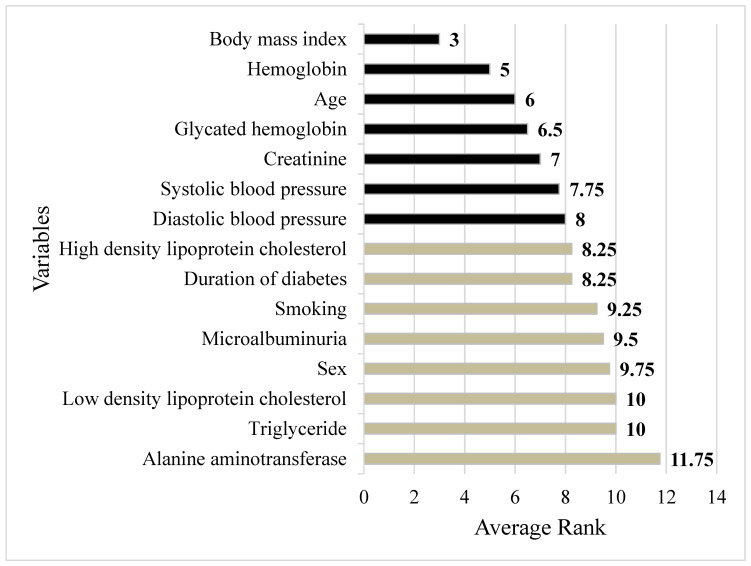
Integrated importance ranking of all risk factors.

Figure 3 shows the risk factors based on the order of their mean ranking values. It can be noted from the figure that the first seven important risk factors in predicting abnormal Th-201 scans are BMI, Hb, age, HbA1c, Cr, SBP and DBP, accordingly.

## 4. Discussion

As aforementioned in the introduction, there are two goals of the present study. Our results show that: 1. All the ML methods are not inferior to traditional MLR; 2. BMI, Hb, age, HbA1c, Cr, SBP and DBP, from the most important to the least important, are the major influencers.

Not surprisingly, BMI is the top risk factor in detecting abnormal Th-201 scans, which suggests that obesity is the most critical clinical parameter to predict myocardial hypoperfusion. Accumulative evidence has shown that BMI is associated with an increased risk of CAD and the occurrence of acute coronary artery syndrome [54,55], and it is also an independent indicator in predicting exercise-induced myocardial ischemia [56]. The actual mechanism underlying obesity and CAD remains to be determined, and several hypotheses have been proposed. For instance, obesity is a central part of metabolic syndrome and is associated with complicated metabolic derangements, including glucose intolerance, dyslipidemia, HTN, dysregulated inflammatory cytokines and endothelial dysfunction, all of which lead to susceptibility to CAD [55,57]. In addition, obesity is also correlated with increased coagulation factor and platelets activation, which could result in a hypercoagulable state, and subsequently contribute to the development of CAD [55].

The role of anemia has already been reported in much research [58,59]. From these studies, three conclusions can be drawn: 1. Anemia affects the prognosis of acute coronary disease [58]; 2. Anemia could alter ST-segment changes [59]; 3. Different methods of transfusion could have different impacts on the prognosis of MI [60]. Among these studies, Cook et al. performed a study to show the precise impact of anemia on MPS. In 195 anemic participants, the mean SSS was higher than that of the normal comparators (6.8 vs. 4.7, *p* < 0.01) [61]. It is not surprising that anemia could worsen epicardial CAD and increase blood viscosity and end-diastolic pressure [62,63].

In the present study, age was ranked as the third most important risk factor in predicting abnormal Th-201 scans. It is known that age itself exerts an essential role in the progressive deterioration of overall cardiovascular function, which increases the risk of CAD in the elderly [64]. From a clinical view of point, advanced age is associated with a high risk of T2DM, HTN, dyslipidemia and renal insufficiency, all of which contribute to the occurrence of CAD. On the other hand, old age links certain molecular mechanisms involving vascular aging, and microvascular and macrovascular remodeling, including oxidative stress, mitochondrial dysfunction, inflammation, endothelial dysfunction, etc. [65,66]. All these clinical and molecular factors could facilitate cardiovascular dysfunction and myocardial ischemia in the elderly.

HbA1c, the traditional long-term glycemic control marker of diabetes, was selected as the fourth important parameter for having an abnormal Th-201 scan. In T2DM, sustained exposure to hyperglycemia could increase the incidence and the severity of macrovascular complications, including CAD [67,68,69]. In the additional 10 years of the original UK Prospective Diabetes Study, the intensive glycemic control group demonstrated a long-term reduction of all-cause mortality [70]. Therefore, intensive glycemic control is needed for T2DM patients to reduce macrovascular complications in clinical settings.

Next, Cr was the fifth predictor in the current study, which implies that renal dysfunction was a significant clinical parameter in assessing CAD. In addition to CAD, T2DM is also considered a strong pathogenic factor for having another major microvascular complication, diabetic nephropathy (DN) [71]. DN is the principal cause of chronic kidney disease (CKD) and end-stage renal disease, which is associated with high morbidity and mortality rate [5]. Declined renal function is found to be associated with an increased risk of death, cardiovascular events and hospitalization in a normal population-based study, which suggests CKD is also an independent indicator for CAD risk and mortality [72].

The last two factors chosen were SBP and DBP. It is well-documented that HTN is an independent risk factor for the occurrence of CAD, and BP reduction in hypertensive patients could significantly reduce CAD incidence and mortality [73]. The pathogenesis of HTN in the development of CAD is extremely complicated, including the effect of high BP as a physical pressure on the vessel wall, triggering and aggravating the atherosclerotic process, inducing endothelial dysfunction, facilitating arterial wall stiffness, subsequently leading to left ventricular hypertrophy and increasing myocardial oxygen consumption, ultimately leading to myocardial ischemia [74].

One may question that why certain risk factors, including the duration of diabetes, LDL, HDL, smoking and microalbuminuria, etc., were ranked as less critical clinical parameters in predicting abnormal Th-201 scans using ML methods. This interesting observation could be attributed to the unique nature of ML which are data-directed and non-parametric models. ML methods can process any nonlinear function and a prior hypothesis describing the characteristics of the data is not required. In addition, despite the fact that the actual relationships among the empirical data could be unclear or difficult to illustrate, ML methods can still catch slight functional correlations between them [75,76,77]. Therefore, it is possible that the duration of diabetes, LDL, HDL, smoking and microalbuminuria, etc., may consist of more linear pattern information and less remarkable nonlinear clues than BMI, Hb, age, HbA1c, Cr, SBP and DBP. Consequently, these variables are graded as the less important risk factors using ML methods.

To the best of our knowledge, despite ML methods being used in certain medical fields, however, no study has been carried out to predict abnormal Th-201 scans using ML methods so far. This study is the first one to identify the most critical indicators in predicting diabetic patients with abnormal Th-201 scans and possible CAD using clinical variables together with ML. In addition to evaluating the importance of risk factors ranked by ML, we also compared ML and MLR at the same time. However, there are still limitations to this study. First, this is a cross-sectional study, and our data are less convincing than those of a longitudinal study. Secondly, there were missing values in certain clinical variables, and collecting complete data on these variables would make our results more valuable. Thirdly, as healthy controls were not included in the study, we cannot compare the differences in risk factors between T2DM and non-T2DM subjects. It would be our future plan to recruit healthy subjects as a control group for further analysis to make our results more reliable and comprehensive. Finally, important medication information including lipid-lowering agents, sodium-glucose cotransporter 2 inhibitors and glucagon-like peptide-1 agonists, etc., which can reduce the CAD risk, was not available for the study. Since we did not collect this information, the effects of the aforementioned medications remain unknown.

In conclusion, our data showed that ML methods were not inferior to traditional MLR and might be more accurate than traditional MLR in predicting abnormal Th-201 scans in T2D patients. We recognized that BMI, Hb, age, HbA1c, Cr, SBP and DBP, from most important to least important, are the most significant factors.

## Figures and Tables

**Table 1:** Variable definition.

Variables	Description	Unit
V1: Sex	Male/Female	-
V2: Age	Patient age	year
V3: Body mass index	Body mass index	Kg/m^2^
V4: Duration of diabetes	Duration of diabetes	year
V5: Smoking	No/Yes	-
V9: Glycated hemoglobin	HbA1c (Glycated hemoglobin)	%
V10: Triglyceride	Triglyceride baseline	mg/dL
V11:High density lipoprotein cholesterol	High-Density Lipoprotein Cholesterol	mg/dL
V12: Low density lipoprotein cholesterol	Low-Density Lipoprotein Cholesterol	mg/dL
V13: Alanine aminotransferase baseline	Alanine aminotransferase	U/L
V14: Creatinine	Creatinine	mg/dL
V6: Systolic blood pressure	Systolic blood pressure	mmHg
V7: Diastolic blood pressure	Diastolic blood pressure b	mmHg
V8: Hemoglobin	Hb	
V15: Microalbuminuria	Urine albumin to creatinine ratio = microalbumin (mg/dL)/urine creatinine(mg/dL)	mg/g

**Table 2:** The Equation of Performance Metrics.

Metrics	Calculation *
SMAPE	$SMAPE=\frac{1}{m}\overset{m}{\underset{j=1}{\sum }}\frac{\left|p_{j}-q_{j}\right|}{\left(\left|p_{j}\right|+\left|q_{j}\right|\right)/2}$
RAE	$RAE=\frac{\sum_{j=1}^{m}\left|p_{j}-q_{j}\right|}{\sum_{j=1}^{m}\left|q_{j}-\bar{q}\right|}$
RRSE	$RRSE=\sqrt{\frac{\sum_{j=1}^{m}{\left(p_{j}-q_{j}\right)}^{2}}{\sum_{j=1}^{m}{\left(q_{j}-\bar{q}\right)}^{2}}}$
RMSE	$RMSE=\sqrt{\frac{1}{m}\overset{m}{\underset{j=1}{\sum }}{\left(p_{j}-q_{j}\right)}^{2}}$

SMAPE, Symmetric Mean Absolute Percentage Error; RAE, Relative Absolute Error; RRSE, Root- Relative Squared Error; RMSE, Root Mean Squared Error. * *p* and *q* represent predicted and actual values, respectively; m is the total number of data.

**Table 3:** The demographics of enrolled type 2 diabetes patients.

Variables	Mean ± SD	N
Age	68.09 ± 10.07	796
Body mass index	26.17 ± 3.89	588
Duration of diabetes	13.81 ± 8.02	589
Fasting plasma glucose	150.09 ± 46.05	591
Glycated hemoglobin	7.68 ± 1.39	590
Triglyceride	123.65 ± 79.32	586
High-density lipoprotein cholesterol	49.53 ± 14.98	524
Low-density lipoprotein cholesterol	95.52 ± 26.18	588
Alanine aminotransferase baseline	23.66 ± 13.60	588
Creatinine	1.16 ± 0.99	587
Systolic blood pressure	131.08± 15.36	514
Diastolic blood pressure	73.35 ± 10.09	514
Microalbuminuria	196.53± 723.55	551
	**N (%)**	**N**
Sex		796
Male	369 (53.64%)	
Female	427 (46.36%)	
Smoking		329
No	212 (64.44%)	
Yes	117 (35.56%)	

SD, standard deviation.

**Table 4:** The performance of multiple linear regression (MLR) and different machine learning methods.

Mean (SD)	SMAPE	RAE	RRSE	RMSE
MLR	1.120(0.04)	1.049(0.06)	1.054(0.03)	7.760(0.39)
RF	1.070(0.03)	1.043(0.05)	1.042(0.02)	7.683(0.48)
SGB	1.074(0.03)	1.026(0.05)	1.039(0.03)	7.661(0.45)
CART	1.055(0.04)	1.031(0.06)	1.049(0.03)	7.736(0.56)
XGBoost	1.058(0.04)	1.017(0.05)	1.032(0.02)	7.613(0.58)

RF, random forest; SGB, stochastic gradient boosting; CART, classification and regression tree; XGBoost, eXtreme gradient boosting; SMAPE, symmetric mean absolute percentage error; RAE, relative absolute error; RRSE, root relative square error; RMSE, root mean square error. The numbers in the parentheses are standard errors.

**Table 5:** Importance ranking of each risk factor using the four convincing methods.

Variables	RF	SGB	CART	XGBoost	Average
Sex	5	14	6	14	9.75
Age	2	4	3	15	6
Body mass index	4	1	1	6	3
Duration of diabetes	1	13	11	8	8.25
Smoking	6	15	15	1	9.25
Hemoglobin	8	6	4	2	5
Glycated hemoglobin	9	2	5	10	6.5
Triglyceride	10	10	8	12	10
High density lipoprotein cholesterol	11	7	10	5	8.25
Low density lipoprotein cholesterol	12	5	12	11	10
Alanine aminotransferase baseline	13	8	13	13	11.75
Creatinine	14	3	2	9	7
Systolic blood pressure	7	11	9	4	7.75
Diastolic blood pressure	3	12	14	3	8
Microalbuminuria	15	9	9	9	9.5

RF, random forest; SGB, stochastic gradient boosting; CART, classification and regression tree; XGBoost, eXtreme gradient boosting.

## Data Availability

Data available on request due to privacy/ethical restrictions.
